# 18F‐fluorodeoxyglucose uptake in PET is associated with the tumor microenvironment in metastatic lymph nodes and prognosis in N2 lung adenocarcinoma

**DOI:** 10.1111/cas.15266

**Published:** 2022-02-18

**Authors:** Kotaro Nomura, Tokiko Nakai, Yukino Nishina, Naoya Sakamoto, Tomohiro Miyoshi, Kenta Tane, Joji Samejima, Keiju Aokage, Motohiro Kojima, Shingo Sakashita, Tetsuro Taki, Saori Miyazaki, Reiko Watanabe, Kenji Suzuki, Masahiro Tsuboi, Genichiro Ishii

**Affiliations:** ^1^ Department of Pathology and Clinical Laboratories National Cancer Center Hospital East Kashiwa Japan; ^2^ Department of Thoracic Surgery National Cancer Center Hospital East Kashiwa Japan; ^3^ Department of General Thoracic Surgery Juntendo University School of Medicine Tokyo Japan; ^4^ Department of Integrated Biosciences Laboratory of Cancer Biology Graduate School of Frontier Sciences The University of Tokyo Kashiwa Japan; ^5^ Division of Pathology Exploratory Oncology Research and Clinical Trial Center National Cancer Center Kashiwa Japan; ^6^ Division of Innovative Pathology and Laboratory Medicine Exploratory Oncology Research and Clinical Trial Center National Cancer Center Kashiwa Japan

**Keywords:** 18F‐fluorodeoxyglucose‐positron emission tomography, cancer‐associated fibroblast, lung adenocarcinoma, lymph node metastasis, prognosis

## Abstract

Positron emission tomography is a useful technique for diagnosing lymph node (LN) metastasis. This study aimed to elucidate the association between fluorodeoxyglucose accumulation and the microenvironment in metastatic LNs in lung adenocarcinoma. We retrospectively analyzed 62 patients with surgically resected pathological N2 lung adenocarcinoma who underwent preoperative PET. The maximum standardized uptake value (SUV_max_) in the metastatic LNs was measured. Lymph node specimens were immunohistochemically analyzed for CD8^+^, FoxP3^+^, and CD79a^+^ lymphocytes, CD204^+^ tumor‐associated macrophages (TAMs), and alpha‐smooth muscle actin‐positive cancer‐associated fibroblasts (αSMA^+^ CAFs). We compared the clinicopathologic and immunohistochemical characteristics between two groups with high and low LN SUV_max_. Using novel 3D hybrid spheroid models, we investigated the change in invasiveness of cancer cells in the presence of CAFs. In the multivariate analyses, LN SUV_max_ was an independent prognostic factor. The overall survival in the LN SUV_max_ high group was significantly worse than in the low group (*P* = .034). In the LN SUV_max_ high group, metastatic cancer cell invasion of extranodal tissue was more frequent (*P* = .005) and the number of CD204^+^ TAMs and αSMA^+^ CAFs in metastatic LNs was significantly higher than in the low group (*P* < .001 and *P* = .002, respectively). Hybrid spheroid models revealed that cancer cells coexisting with CAFs were more invasive than those without CAFs. Our results indicated a strong association between LN SUV_max_ and poor prognosis in patients with N2 lung adenocarcinoma. Moreover, LN SUV_max_ was suggested to be associated with the presence of tumor‐promoting stromal cells in metastatic LNs.

## Graphical abstract

18F‐fluorodeoxyglucose uptake in metastatic lymph nodes (LNs) in PET was strongly associated with poor prognosis in patients with N2 lung adenocarcinoma. Moreover, high LN maximum standardized uptake value was associated with the presence of cancer‐associated fibroblasts and tumor‐associated macrophages in the metastatic LNs.

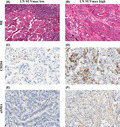

## 1 INTRODUCTION

Mediastinal lymph node (LN) metastasis (N2) is one of the most important poor prognostic factors in patients with non‐small‐cell lung cancer (NSCLC). Despite recent advances in perioperative treatment, the prognosis in patients with surgically resected pathological N2 NSCLC remains poor.^1^ Some researchers have mentioned that the prognosis in this population is heterogeneous because of various patterns of LN metastasis, such as the number of metastatic LNs or the presence of extranodal extension (ENE).^2^, ^3^, ^4^

18F‐fluorodeoxyglucose (FDG)‐PET is a useful imaging technique for staging patients with NSCLC. 18F‐fluorodeoxyglucose‐PET provides images based on the accumulation of glucose in malignant cells^5^ and helps in the diagnosis of metastases. Furthermore, FDG uptake has been reported to represent disease activity and prognosis in patients with NSCLC.^6^, ^7^ High FDG uptake in primary tumors is associated with high tumor invasiveness and poor patient prognosis.^8^ Previous studies have reported that tumor‐promoting stromal cells, such as cancer‐associated fibroblasts (CAFs) and tumor‐associated macrophages (TAMs) are abundant in primary tumors of lung adenocarcinoma patients with high FDG uptake.^9^ Thus, FDG uptake in the primary tumor reflects the biological behavior of the primary tumor microenvironment. However, few studies have investigated the association between the tumor microenvironment in metastatic LNs and FDG uptake.

Although FDG uptake in PET provides biological information on cancer cells, reports on FDG uptake in metastatic LNs are lacking. We hypothesized that FDG uptake in metastatic LNs is associated with microenvironmental factors in these LNs, similar to what was observed in primary tumors, and predicts the prognosis in patients with pathological N2 lung adenocarcinoma. This study aimed to elucidate the association between FDG uptake in metastatic LNs and the prognosis in patients with surgically resected pathological N2 lung adenocarcinoma. Furthermore, we investigated the association between FDG uptake and the tumor microenvironment in metastatic LNs.

## 2 MATERIALS AND METHODS

### 2.1 Patients

A total of 62 patients were included in the study. A flowchart of the patient selection process is presented in Figure S1. This retrospective study was approved by the institutional review board of the National Cancer Center Hospital East (approval no. 2020‐147). Comprehensive informed consent was obtained from all patients.

#### 2.1.1 Positron emission tomography/computed tomography imaging protocol

As detailed in previous studies,^9^, ^10^ all 18F‐FDG‐PET imaging procedures were carried out using Discovery LS, STELite, or IQ (GE Healthcare). Patients were injected with 240‐300 MBq of 18F‐FDG after fasting for at least 6 hours to minimize the serum glucose level. Sixty minutes after the injection of FDG, the PET scan was carried out using a 2D acquisition mode from the skull to the thigh with seven bed positions. Attenuation correction of the PET images was undertaken using data from the computed tomography (CT) images. The PET images were reconstructed using an ordered‐subset expectation maximization algorithm (two iterations and 14 subsets in Discovery LS, two iterations and 21 subsets in STELite, and three iterations and 12 subsets in IQ). The reconstructed images were evaluated using the standardized uptake value (SUV). To determine the SUV, a round region of interest was manually drawn at primary tumor or metastatic LN sites on the axial slice. The FDG accumulation was determined as the SUV, and the maximum SUV (SUV_max_) was obtained (Figure S2).

### 2.2 Histological evaluation

All surgical specimens were fixed in 10% formalin, embedded in paraffin, and serially sectioned at 4‐µm intervals. Two pathologists (K.N. and T.N.) evaluated all stained tissue sections under a light microscope. Histological typing was based on the 4th edition of the WHO histological classification^11^ and the disease stages were categorized according to the guidelines provided in the 8th edition of the UICC TNM classification.^12^

### 2.3 Immunohistochemical staining and immunohistochemical scoring

Among the 62 patients, we undertook immunohistochemical staining for LN specimens obtained from 59 patients (specimens for three patients were not available; Figure S3). Immunohistochemical staining was carried out using the Benchmark ULTRA system (Ventana, Roche) with the primary Abs listed in Table S1. All slides containing LNs immunohistochemically stained with each Ab were scanned using Aperio AT2 (Leica Biosystems). The number of FoxP3^+^, CD8^+^, or CD79a^+^ lymphocytes, and CD204^+^ TAMs were manually counted in five randomly selected independent areas (0.0625 mm^2^/field) containing metastatic tumor cells. The average number of positive cells in each field was calculated and recorded. Glucose transporter‐1 (GLUT‐1) immunostaining was scored according to previous reports.^9^, ^13^ The staining intensity was scored on a three‐tier scale from 0 (absent) to 2 (strong staining); these were multiplied by the percentages of immunohistochemically stained tumor cells, resulting in immunostaining scores ranging from 0 to 200. The area of alpha‐smooth muscle actin (αSMA)^+^ CAFs (mm^2^) was automatically calculated using the analysis function (Aperio Image Scope version 12.3.3; Leica Biosystems Imaging).

### 2.4 Cell culture

The human lung adenocarcinoma cell line A549 was obtained from ATCC. A549 cells labeled with monomeric red fluorescent protein (RFP)^14^ were cultured in DMEM F‐12 Ham (Sigma‐Aldrich), supplemented with 10% FBS (Thermo Fisher Scientific), and 1% penicillin and streptomycin (Sigma). The cells were incubated at 37°C in an atmosphere containing 5% CO_2_.

Cancer‐associated fibroblasts were prepared from human lung adenocarcinoma tissues as previously described.^15^ The institutional review board of the National Cancer Center Hospital East approved this study (approval no. 2005‐043).

### 2.5 Lifetime extension of CAFs

To extend the lifetime of CAFs, transduction was undertaken using a combination of human telomerase reverse transcriptase and mutant forms of cyclin‐dependent kinase 4 (hTERT/CDK4R24C) according to a previously reported method.^15^ The CAFs with extended lifespan expressed Venus, a fluorescent protein.

### 2.6 Generation and evaluation method of 3D hybrid cancer spheroids

The method for generating 3D hybrid cancer spheroids is shown in Figure S4. First, we prepared two types of cell mixtures: (a) 1.0 × 10^4^ A549 cells only; and (b) 0.1 × 10^4^ A549 cells + 0.9 × 10^4^ CAFs with extended lifespan. Finally, 1.0 × 10^4^ of cell mixtures was seeded onto 96‐well low attachment plates (Sumitomo Bakelite) and incubated overnight at 37°C. The next day, the medium was removed and the cell aggregates were embedded in 50 µL collagen (Cellmatrix Type Ⅰ‐A; Nitta Gelatin) and incubated for 30 minutes at 37°C in an atmosphere containing 5% CO_2_. After the polymerization of collagen was confirmed, 100 µL medium (DMEM F‐12 Ham : α‐MEM = 1:1) was added on the top of the collagen. The evaluation method for the number of cancer cells invading the collagen gel discontinuously from the main spheroid is described in Figure S5. First, we encircled the edge of the main spheroid on day 6 of culture on the H&E‐stained image (orange solid line in Figure S5). We calculated the average distance between the center of gravity and edge of the cell cluster (average of the center of gravity‐distance). We drew a circle with the center of gravity of the cell cluster as a center and a radius of three times the average of the center of gravity‐distance (orange dotted circle in Figure S5). Next, the circle on the H&E‐stained image was reflected on the fluorescence immunostaining image. We measured the number of cancer cells invading the collagen gel discontinuously from the main spheroid in the circle. For this analysis, we used the image analysis software WinROOF version 6.5 (MITANI Corporation).

### 2.7 Immunofluorescence analysis of 3D hybrid cancer spheroids

Hybrid cancer spheroids were fixed with 10% formalin, embedded in paraffin, and serially sectioned at 4‐µm intervals. Slides were incubated with primary Abs (anti‐GFP Ab, chicken polyclonal and anti‐RFP Ab, rabbit polyclonal [both Abcam]) at 4°C overnight. After washing with PBS, slides were incubated with secondary immunofluorescent Ab (Alexa Fluor 546 goat anti‐chicken Ab and Alexa Fluor 488 donkey anti‐rabbit Ab, respectively) at 4°C for 1 hour. For nuclear staining, slides were incubated with DRAQ5 (BioStatus) at 4°C for 15 minutes.

### 2.8 Survival statistical analysis

The length of overall survival (OS) was defined as the period between the date of surgery and the last follow‐up date or death due to any cause. The length of recurrence‐free survival (RFS) was defined as the period between the date of surgery and the date of last follow‐up, the first recurrence, or death due to any cause. The data cut‐off date was January 31st 2021 at our institution.

Overall survival and RFS curves were plotted according to the Kaplan‐Meier method and compared using the log‐rank test in a univariate analysis. To determine the predictors of OS and RFS, univariate and multivariate analyses were undertaken using Cox regression analysis. Two‐category comparison was carried out using the Wilcoxon rank sum test for continuous variables and Fisher’s exact test for categorical variables. All the *P* values were two‐sided, and the statistical significance level was set at *P* < .05. All statistical analyses were carried out using SPSS version 27.0 software (SPSS Inc.).

## 3 RESULTS

### 3.1 Prognostic significance of SUV_max_ in LNs on PET/CT

The clinical and pathological characteristics of the 62 patients are shown in Table S2. Table 1 shows the results of univariate and multivariate Cox regression analyses of OS in all patients. Univariate Cox regression analysis of OS indicated that male sex (*P* = .015), increase in the SUV_max_ in metastatic LNs (LN SUV_max_; *P* = .002), and history of adjuvant chemotherapy (*P* = .016) were significant prognostic factors. Multivariate Cox regression analysis revealed that these factors were significantly and independently associated with patient prognosis. Regarding RFS, the univariate Cox regression analysis showed that an increase in the LN SUV_max_ was a significant prognostic factor (*P* = .021; Table S3).

**TABLE 1:** Univariate and multivariate analyses for overall survival in pN2 lung adenocarcinoma patients

Variable	Univariate			Multivariate		
	HR	95% CI	*P* value	HR	95% CI	*P* value
Age						
+1	1.012	0.973‐1.053	.541	‐	‐	‐
Sex, male						
Female	Ref.	‐	‐	‐	‐	‐
Male	2.741	1.214‐6.186	.015	3.046	1.319‐7.034	.009
Smoking history						
Never	Ref.	‐	‐	‐	‐	‐
Ever	1.740	0.812‐3.727	.154	‐	‐	‐
CEA						
+1 ng/mL	0.995	0.982‐1.007	.404	‐	‐	‐
SUV_max_						
Primary, +1	1.029	0.940‐1.126	.540	‐	‐	‐
LN, +1	1.184	1.061‐1.321	.002	1.142	1.026‐1.270	.015
Invasive size						
+1 cm	1.078	0.880‐1.322	.467	‐	‐	‐
Lymphatic invasion						
Absent	Ref.	‐	‐	‐	‐	‐
Present	1.345	0.657‐2.755	.418	‐	‐	‐
Pleural invasion						
Absent	ref.	‐	‐	‐	‐	‐
Present	1.693	0.802‐3.573	.167	‐	‐	‐
Tumor size in LN						
+1 mm	1.061	0.997‐1.130	.063	‐	‐	‐
Number of N2 stations						
Single	ref.	‐	‐	‐	‐	‐
Multiple	0.976	0.469‐2.030	.948	‐	‐	‐
*EGFR* mutation						
Absent	Ref.	‐	‐	‐	‐	‐
Present	0.716	0.345‐1.487	.370	‐	‐	‐
Adjuvant chemotherapy						
Absent	Ref.	‐	‐	‐	‐	‐
Present	0.408	0.197‐0.845	.016	0.353	0.166‐0.750	.007

Abbreviations: –, not included in analysis; CEA, carcinoembryonic antigen; CI, confidence interval; EGFR, epidermal growth factor receptor; HR, hazard ratio; LN, lymph node; Ref., reference; SUV_max_, maximum standardized uptake value.

**FIGURE 1:**
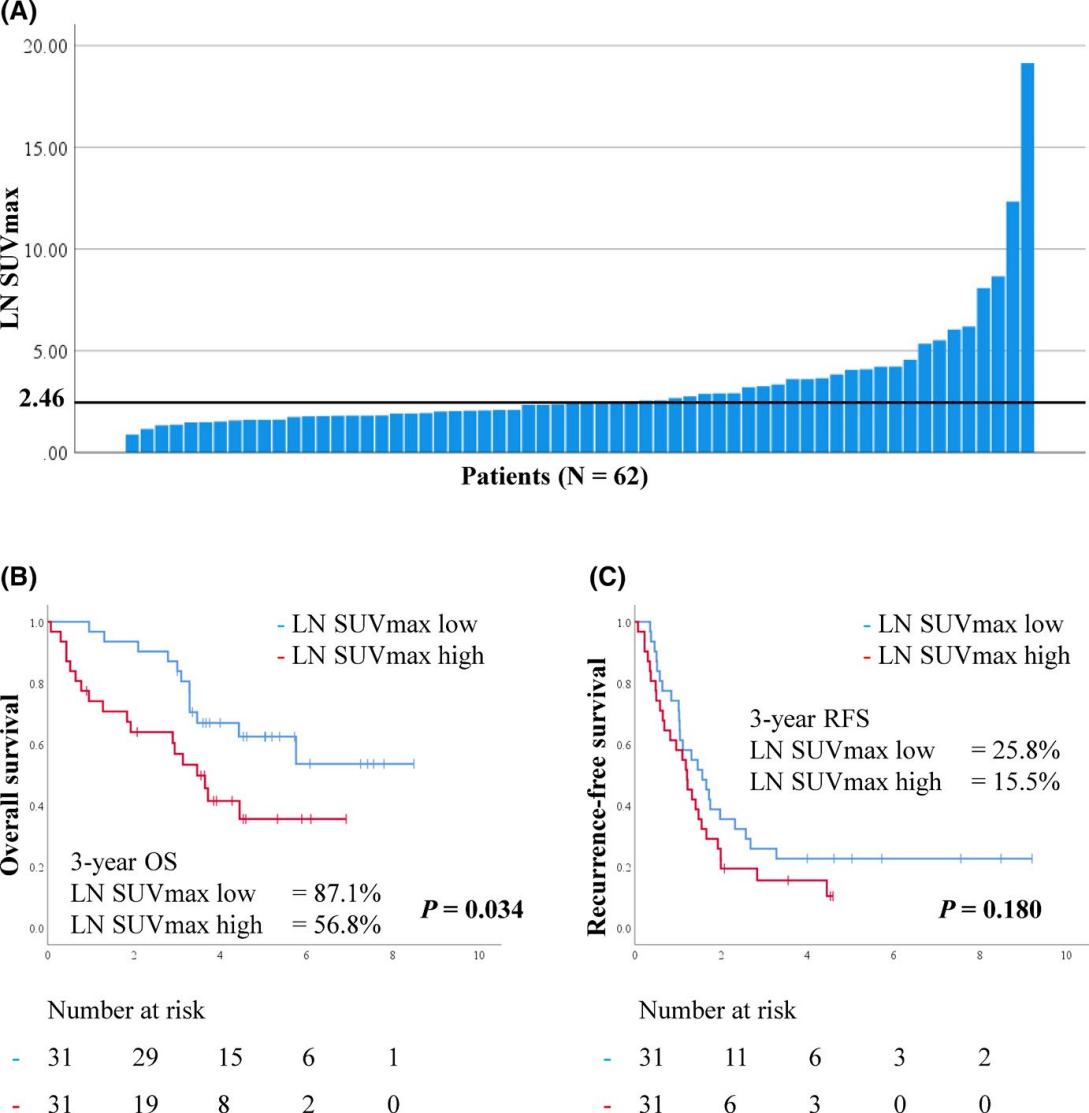
A, Histogram showing maximum standardized uptake values (SUV_max_) in metastatic lymph node (LN) in 62 patients with pN2 lung adenocarcinoma. The median LN SUV_max_ is 2.46. B, C, Kaplan‐Meier curves showing overall survival (OS) (B) and recurrence‐free survival (RFS) (C) in patients according to LN SUV_max_

Furthermore, we divided all patients into two categories based on LN SUV_max_. We considered patients with LN SUV_max_ ≥ median value (2.46) as the LN SUV_max_ high group (n = 31), and those with LN SUV_max_ < median as the LN SUV_max_ low group (n = 31; Figure 1A). The Kaplan‐Meier curves showed that OS was significantly shorter in the LN SUV_max_ high group than in the LN SUV_max_ low group (*P* = .034). The 3‐year OS in the LN SUV_max_ high and low groups was 56.8% and 87.1%, respectively (Figure 1B). The 3‐year RFS in the LN SUV_max_ high and low groups was 15.5% and 25.8%, respectively. However, RFS was not significantly different between the LN SUV_max_ high and low groups (*P* = .180; Figure 1C).

### 3.2 Clinicopathologic difference between LN SUV_max_ high and low groups

**FIGURE 2:**
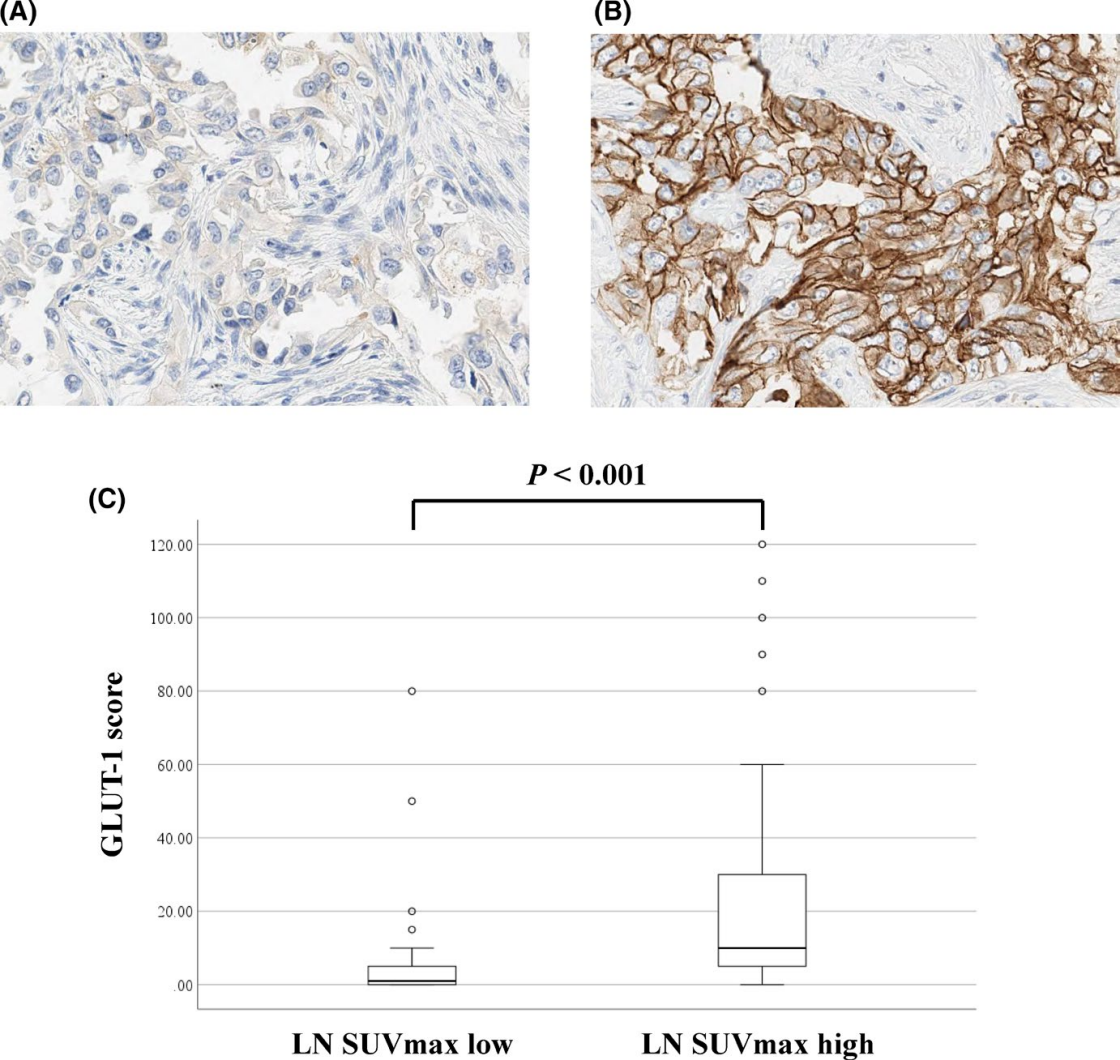
Immunohistochemical staining and scoring for glucose transporter‐1 (GLUT‐1) in metastatic lymph node (LN) in patients with pN2 lung adenocarcinoma. A, B, Immunohistochemical staining for GLUT‐1 in LN with low (A) or high (B) maximum standardized uptake value (SUV_max_). C, Immunohistochemical score of GLUT‐1 in metastatic LN according to the LN SUV_max_

Tables 2 and S4 present the clinicopathologic differences between the LN SUV_max_ high and low groups. In the LN SUV_max_ high group, the primary tumor also showed higher SUV_max_ than that in the LN SUV_max_ low group (*P* = .050). Pathologically, the median invasive tumor size in the primary lesion and tumor size in the metastatic LN in the LN SUV_max_ high group were significantly larger than those in the LN SUVmax low group (*P* = .023 and *P* < .001, respectively). ENE was significantly more frequent in the LN SUV_max_ high group (Figures S6A,B) than in the LN SUV_max_ low group (Figures S6C,D; *P* = .005, Table 2). Immunohistochemically, the GLUT‐1 scores of metastatic LNs in the LN SUV_max_ high group (Figure 2A) were significantly higher than those in the LN SUV_max_ low group (Figure 2B,C; *P* < .001). This result was in line with previous reports on the association between FDG uptake and GLUT‐1 expression in primary lung adenocarcinoma.^9^, ^16^

**TABLE 2:** Clinicopathologic differences between high and low maximum standardized uptake values (SUV_max_) in metastatic lymph node (LN SUV_max_) in patients with pN2 lung adenocarcinoma

Characteristic	LN SUV_max_ low (<2.46) n = 31	LN SUV_max_ high (≥2.46) n = 31	*P* value
Age, years	65 (48‐84)	68 (42‐86)	.041
Sex			
Male	15 (48)	21 (68)	.198
Female	16 (52)	10 (32)	
Smoking history			
Never smoker	15 (48)	10 (32)	.300
Ever smoker	16 (52)	21 (68)	
CEA, ng/mL	6.0 (1.1‐380.5)	6.0 (1.2‐56.9)	.746
Tumor size in LN, mm	3.3 (0.5‐13.6)	8.5 (1.0‐21.5)	<.001
Metastatic N2 stations			
Single	15 (48)	21 (68)	.198
Multiple	16 (52)	10 (32)	
Extranodal extension			
Absent	22 (71)	10 (32)	.005
Present	9 (29)	21 (68)	

Note

Data are shown as n (%) or median (range).

Abbreviations: CEA, carcinoembryonic antigen; LN, lymph node; SUV, standardized uptake value.

### 3.3 Correlations between LN SUV_max_ and tumor microenvironment in metastatic LNs

**FIGURE 3:**
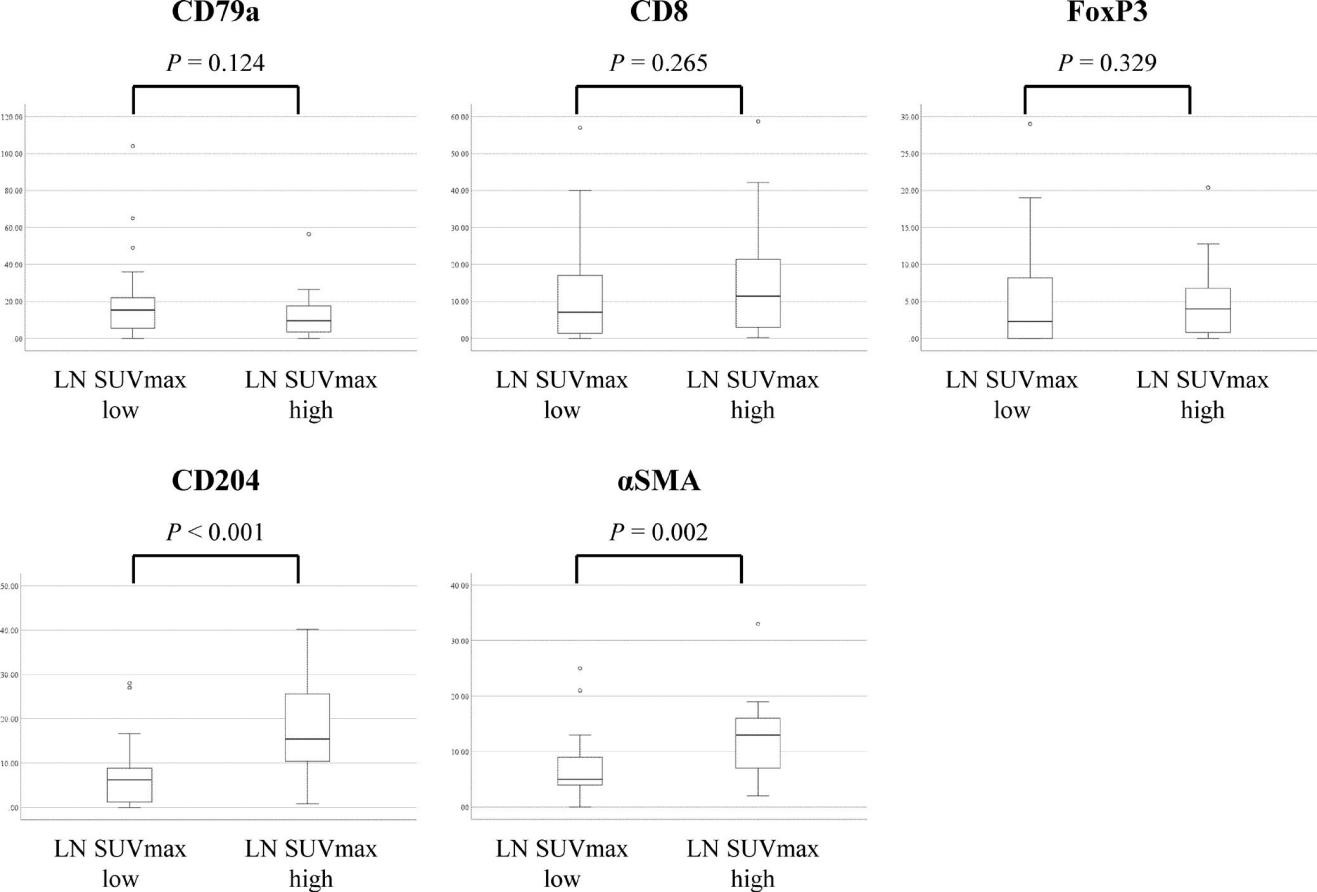
Number of CD79a^+^, CD8^+^, and FoxP3^+^ lymphocytes, CD204^+^ macrophages, and the area of alpha smooth muscle actin (αSMA)^+^ cancer‐associated fibroblasts in metastatic lymph node (LN) in patients with pN2 lung adenocarcinoma, according to the LN maximum standardized uptake value (SUV_max_)

**FIGURE 4:**
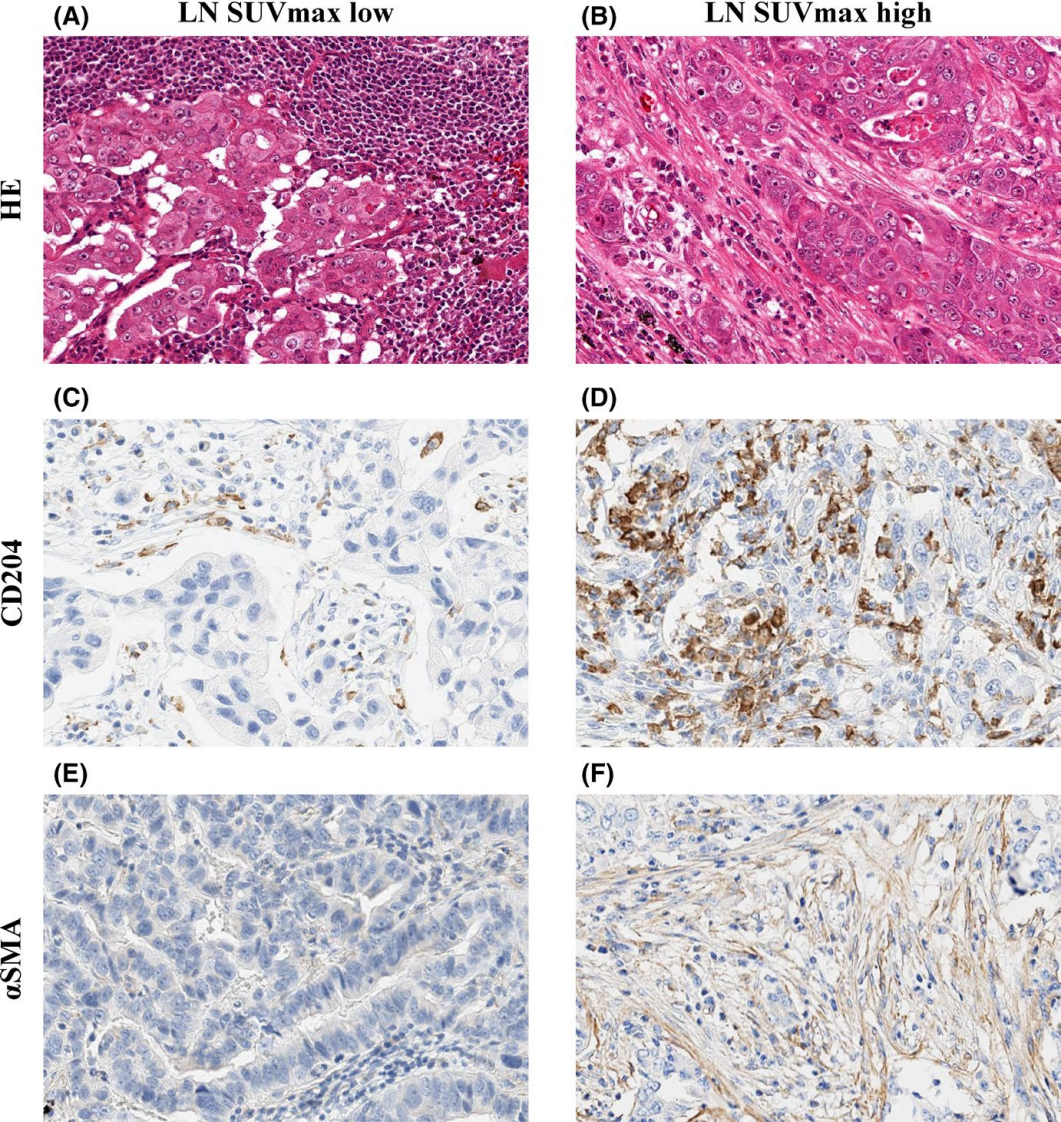
Hematoxylin‐eosin staining (A, B) and immunohistochemical staining for CD204 (C, D), and alpha smooth muscle actin (αSMA) (E, F) in metastatic lymph node (LN) in patients with pN2 lung adenocarcinoma, according to the LN maximum standardized uptake value max (SUV_max_)

The immunohistochemical scores of CD204^+^ TAMs and αSMA^+^ CAFs in metastatic LNs in the LN SUV_max_ high group were significantly higher than those in the LN SUV_max_ low group (*P* < .001 and *P* = .002, respectively). In contrast, the immunohistochemical scores of CD79a, CD8, and FoxP3 in metastatic LNs were not significantly different (Figure 3). Figure 4 shows representative H&E staining images (Figure 4A,B), immunohistochemical staining with CD204 (Figure 4C,D), and αSMA (Figure 4E,F) in the LN SUV high and low groups.

### 3.4 Correlation between CAFs and cancer cell invasion in 3D spheroid model

**FIGURE 5:**
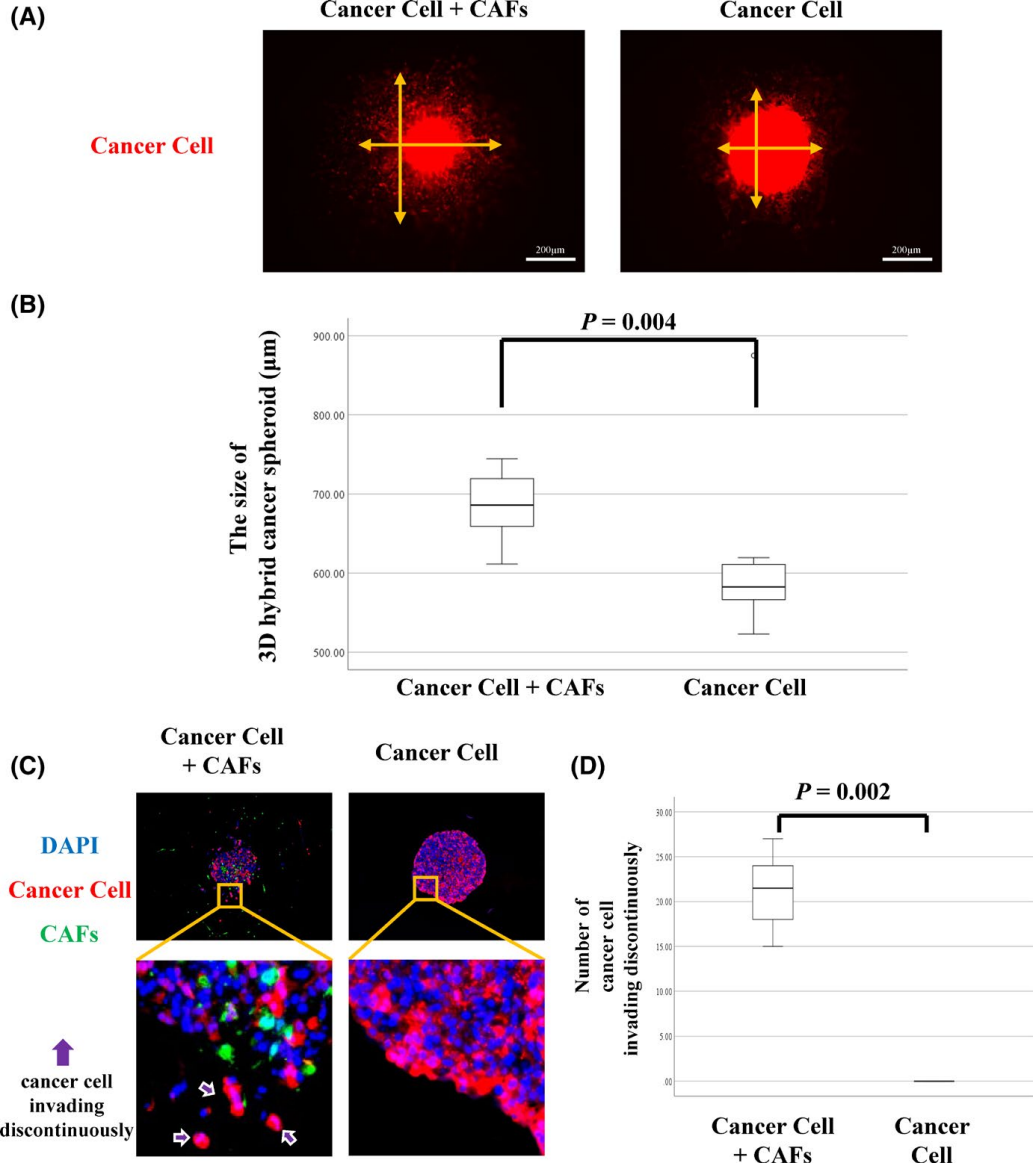
A, Representative images of 3D hybrid cancer spheroid with or without cancer‐associated fibroblasts (CAFs). Orange arrows are vertical and horizontal axes of spheroids. B, Size of 3D hybrid cancer spheroids depending on the proportion of CAFs. C, Representative images of 3D hybrid cancer spheroid with or without CAFs (red, cancer cell; green, CAF; blue, nucleus). D, Number of cancer cells invading into the collagen gel discontinuously from the main spheroid according to the proportion of CAFs

We generated a novel 3D hybrid cancer spheroid and examined how CAFs acted in the metastatic LN tumor microenvironment. Figure 5A shows representative fluorescent images of cancer spheroids with and without CAFs. The median size of cancer spheroids with CAFs (752 μm; range, 626‐833 μm) was significantly larger than that of spheroids without CAFs (603 μm; range, 563‐878 μm) (*P* = .004, Figure 5B). Figure 5C shows representative immunofluorescence staining images of cancer cells invading the collagen gel discontinuously from the main spheroid (purple arrows). The median number of cancer cells was 21.5 (range, 15‐27) in the presence of CAFs. In contrast, no cancer cells invading collagen gel discontinuously from the main spheroid was observed in the absence of CAFs (*P* = .002; Figure 5D).

## 4 DISCUSSION

In this study, we have shown that high FDG uptake in metastatic LNs predicts poor prognosis in patients with pN2 lung adenocarcinoma. Histologically, metastatic cancer cell invasion of ENE tissue was more frequent in metastatic LNs with high FDG uptake. Furthermore, tumor promoting stromal cells, αSMA^+^ CAFs, and CD204^+^ TAMs, were recruited more frequently to metastatic LNs with high FDG uptake. The hybrid spheroid models revealed that the invasiveness of cancer cells was promoted in the presence of CAFs. To our knowledge, this is the first study to investigate the correlation between FDG uptake and the microenvironment in metastatic LNs.

Several researchers have previously reported an association between FDG uptake in primary lung cancer in PET and glucose metabolic markers such as GLUT‐1 or the sodium‐glucose cotransporter family.^16^, ^17^, ^18^ Furthermore, previous studies have shown that high FDG uptake in primary lung cancer, reflecting high glycolytic metabolism, was associated with poor prognosis.^6^, ^8^, ^9^ In the current study, we showed that FDG uptake in metastatic LNs, not primary tumors, was an independent prognostic factor in patients with N2 lung adenocarcinoma. These results suggest that FDG uptake in metastatic LNs is more useful for predicting patient prognosis than that in primary tumors.

Cancer‐associated fibroblasts in primary tumors have been reported to be involved in the invasiveness of cancer cells^19^ and the prognosis in patients with lung adenocarcinoma by several studies.^20^, ^21^, ^22^ In breast cancer, CAFs in metastatic LNs also stimulate cancer cell migration, initiate epithelial‐to‐mesenchymal transition, and are associated with poor prognosis in patients.^23^ In the current study, we carried out an experiment using a hybrid spheroid‐containing collagen gel with cancer cells and CAFs. We showed that the size of the cancer spheroid and the number of cancer cells invading the collagen gel discontinuously from the main spheroid was dramatically increased in the presence of CAFs. Furthermore, this phenomenon was similar to that observed in cancer cells infiltrating the capsule of the metastatic LNs with ENE. These findings suggest that CAFs promote cancer cell invasion and ENE in metastatic LNs. Several studies have reported that TAMs promote cancer cell invasion and migration in primary lung cancer.^24^, ^25^ Thus, CAFs and TAMs play a tumor‐promoting role in the metastatic LN tumor microenvironment.

Recent studies have reported an association between FDG uptake, stromal cells, and cancer cells. Cancer‐associated fibroblasts as well as cancer cells have been reported to show a strong increase in glucose uptake, and thus are detected by FDG PET,^26^ whereas TAMs have been reported to enhance glycolytic metabolism.^27^ These reports support our results that metastatic LNs with high FDG uptake showed abundance in CAFs and TAMs. The association between FDG uptake in metastatic LNs and CAFs and TAMs could explain why high FDG uptake in metastatic LNs predicts poor prognosis in patients with N2 lung adenocarcinoma.

In this study, we revealed that metastatic LNs with high FDG uptake in PET frequently have ENE. We and several researchers previously showed that ENE in metastatic LNs was an important prognostic factor in patients with lung adenocarcinoma.^3^, ^4^ Our results suggest that the FDG uptake in metastatic LNs in PET helps to predict ENE preoperatively.

This study has several limitations. First, this was a retrospective study using a database from a single institute. Second, we did not include patients with N1 lung adenocarcinoma because the SUV_max_ on hilar LNs is difficult to measure due to physiological uptake in the pulmonary artery. Third, the majority of patients in this study were cN0‐1 and confirmed to have pN2 postoperatively. However, this study also included some cN2 and pN2 patients who could not receive definitive chemoradiotherapy due to complications. To remove this bias, a larger, prospective study is required.

In conclusion, we showed an association between FDG uptake in metastatic LNs and prognosis in patients with N2 lung adenocarcinoma. Furthermore, we revealed that FDG uptake in metastatic LNs was associated with a tumor‐promoting microenvironment in LNs. This study suggests that PET is a useful noninvasive tool for predicting patient prognosis and assessing the tumor microenvironment in metastatic LNs.

## DISCLOSURE

The authors have no conflict of interest.

## Supporting information

Fig S1‐S6

Table S1‐S4
