# Supplementary material for: 18F‐fluorodeoxyglucose uptake in PET is associated with the tumor microenvironment in metastatic lymph nodes and prognosis in N2 lung adenocarcinoma
Source: Cancer Sci. 2022 Feb 18;113(4):1488–96. doi: 10.1111/cas.15266 (PMC8990723; doi:10.1111/cas.15266)
Supplement: Supplementary file 1 — Fig S1‐S6 [file CAS-113-1488-s001.docx]

**Supplementary Figures**

**Figure S1.** Patient inclusion flowchart.

**Figure S2.** Representative coronal slice image of contrast enhanced computed tomography.

**Figure S3.** Immunohistochemical staining and immunohistochemical scoring.

**Figure S4.** Schematic representation of the 3D hybrid cancer spheroids methodology.

**Figure S5** Evaluation method for the number of　cancer cells invading into the collagen gel discontinuously from the main spheroid.

**Figure S6.** Hematoxylin-Eosin staining images of metastatic lymph nodes (LNs).


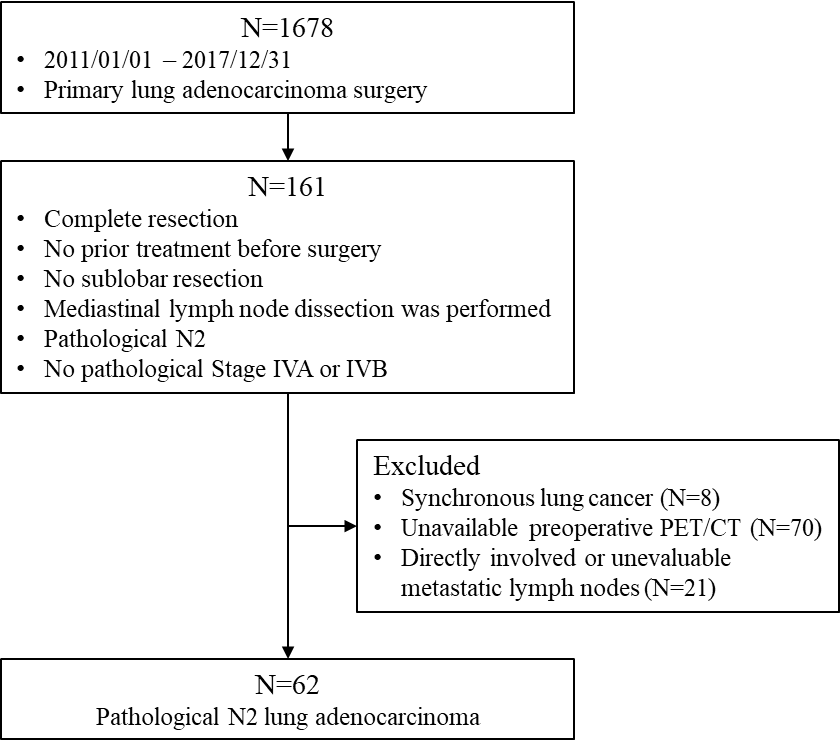


**Figure S1.** Patient inclusion flowchart.

**
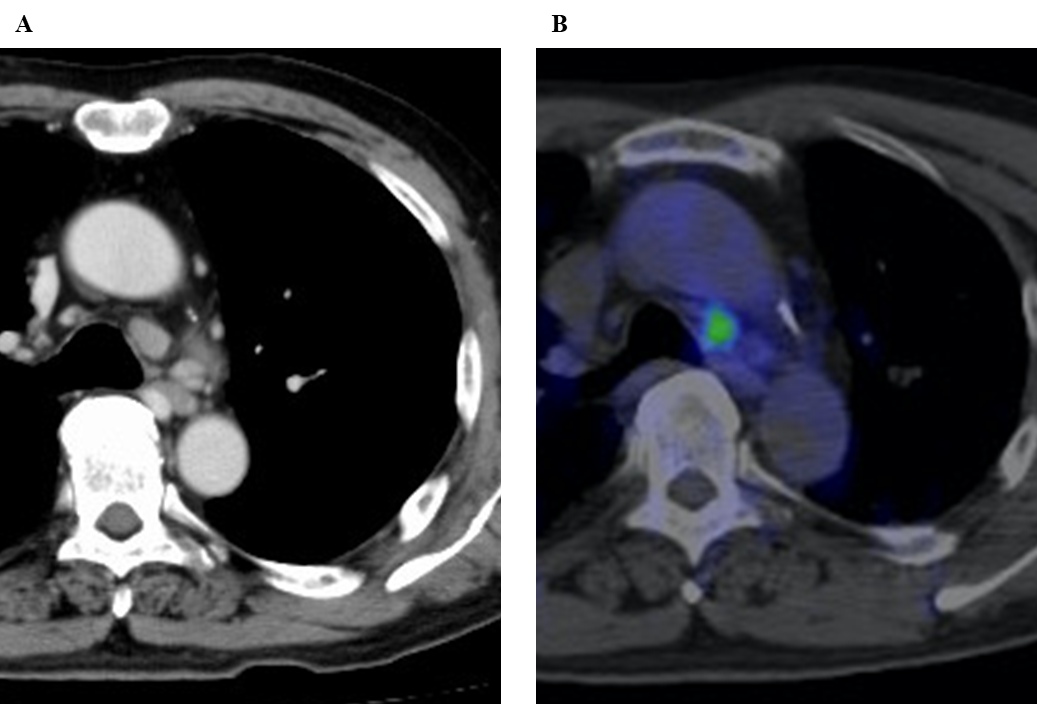
**

**Figure S2.** Representative coronal slice image of contrast enhanced computed tomography (**A**) and integrated positron emission tomography/computed tomography (**B**) in patients with pN2 lung adenocarcinoma.


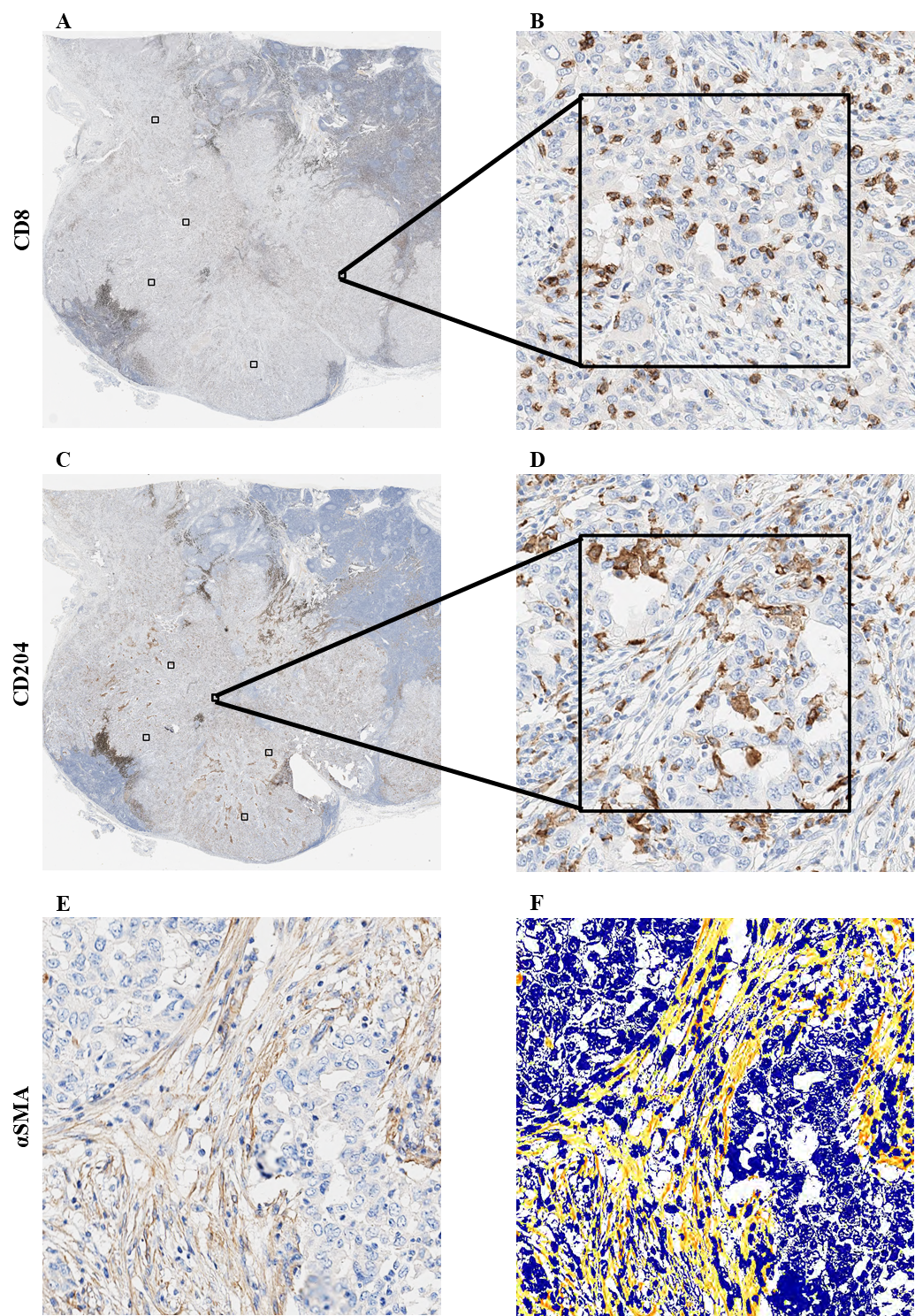


**Figure S3.** Immunohistochemical (IHC) staining for CD8 (**A, B**), CD204 (**C, D**). **A – D** The number of IHC staining-positive cells in randomly chosen five 0.0625mm^2^ squares (black squares) were counted. (**E)** IHC staining for αSMA. (**F)** The areas of αSMA-positive cancer-associated fibroblasts are automatically calculated using Aperio Image Scope.


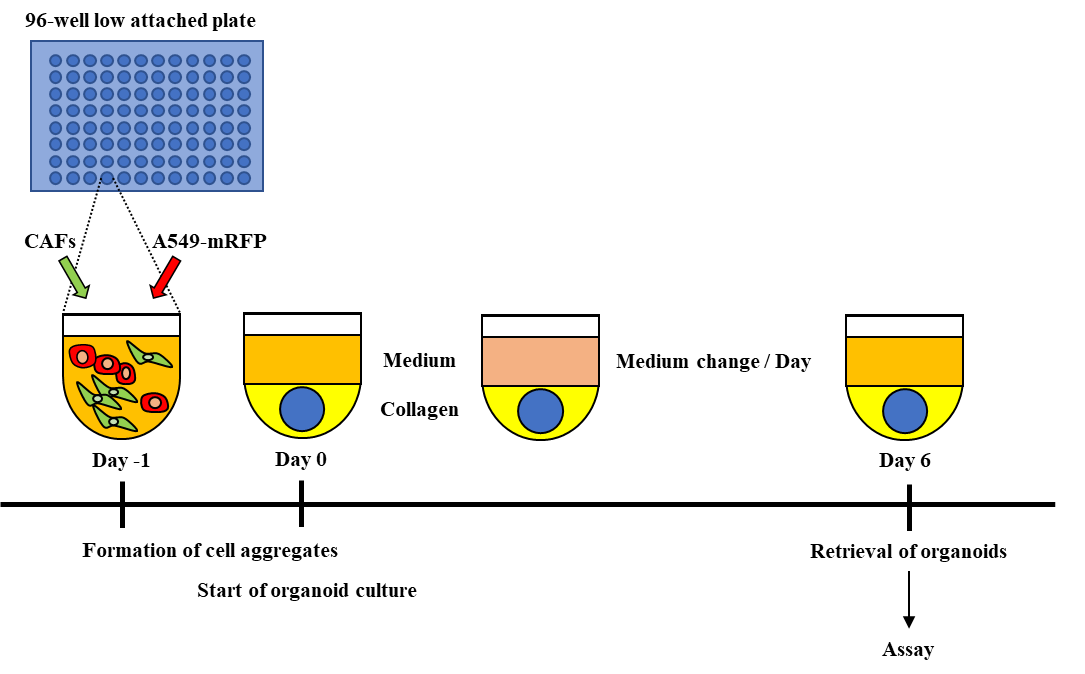


**Figure S4.** Schematic representation of the 3D hybrid cancer spheroids methodology.


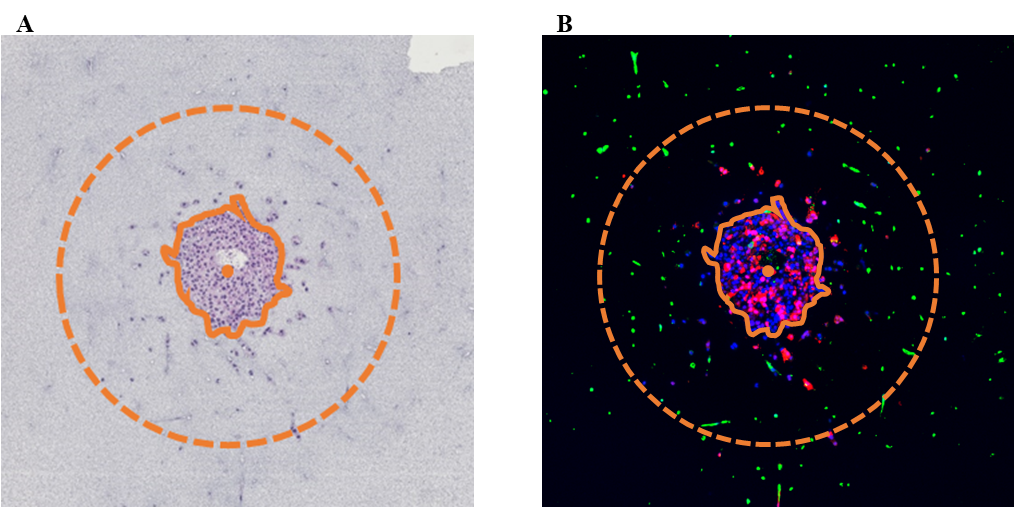


**Figure S5** Evaluation method for the number of　cancer cells invading into the collagen gel discontinuously from the main spheroid. (**A)** Spheroid on HE image. (**B)** Spheroid on fluorescent immunostaining imaging (red, cancer cell; green, CAFs; blue, nucleus).


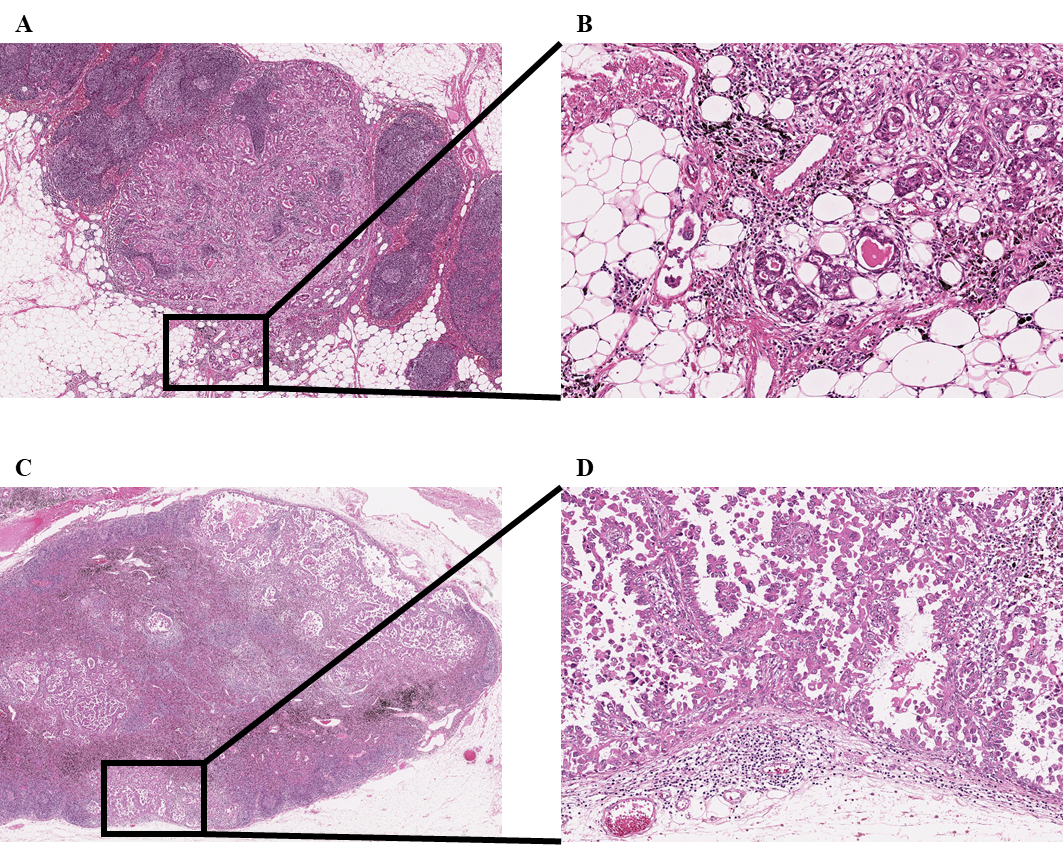


**Figure S6.** Hematoxylin-Eosin staining images of metastatic lymph nodes (LNs). (**A, B)** LN with extranodal extension (ENE) and high SUV_max_ in PET (**A** Lower power view, **B** Higher power view). (**C, D)** LN without ENE and with low SUV_max_ in PET (**C** Lower power view, **D** Higher power view).
