# Supplementary material for: 18F‐fluorodeoxyglucose uptake in PET is associated with the tumor microenvironment in metastatic lymph nodes and prognosis in N2 lung adenocarcinoma
Source: Cancer Sci. 2022 Feb 18;113(4):1488–96. doi: 10.1111/cas.15266 (PMC8990723; doi:10.1111/cas.15266)
Supplement: Supplementary file 2 — Table S1‐S4 [file CAS-113-1488-s002.docx]

**Supplementary Tables.**

**Table S1.** Antibodies for immunohistochemical staining.

**Table S2.** Patient’s clinicopathological characteristics.

**Table S3.** Univariate and multivariate analyses for recurrence-free survival in pN2 patients.

**Table S4.** Clinicopathological differences in primary tumor between LN SUV max high and low.

**Table S1.** Antibodies for immunohistochemical staining.

| Antibody | Clone | Host | Source | Dilution |
| --- | --- | --- | --- | --- |
| Foxp3 | D2W8E | rabbit | Cell Signaling Technology | 1:100 |
| CD8 | C8/468+C8/144B | mouse | abcam | 1:400 |
| CD79a | JCB117 | mouse | DAKO | 1:100 |
| CD204 | SRA-E5 | mouse | Trans Genic Inc. | 1:1000 |
| α-SMA | polyclonal | rabbit | Proteintech Group Inc. | 1:2000 |
| GLUT-1 | polyclonal | rabbit | Proteintech Group Inc. | 1:2000 |

**Table S2.** Patient’s clinicopathological characteristics.

| Characteristic | N=62 | |
| --- | --- | --- |
| Age, median [range] | 67 | [42 – 86] |
| Sex, male (%) | 36 | (58) |
| Smoking history, ever (%) | 37 | (60) |
| CEA, ng/ml, median [range] | 6.0 | [1.1 – 380.5] |
| SUV max, median [range] |  |  |
| primary | 7.41 | [1.69 – 18.11] |
| LN | 2.46 | [0.89 – 19.14] |
| Invasive size, cm, median [range] | 2.5 | [0.9 – 8.0] |
| Lymphatic invasion, + (%) | 28 | (45) |
| Vascular invasion, + (%) | 62 | (100) |
| Pleural invasion, + (%) | 36 | (57) |
| Tumor size in LN, mm, median [range] | 5.8 | [0.5 – 21.5] |
| N2, multiple (%) | 26 | (42) |
| EGFR mutation, + (%), N=57 | 32 | (56) |
| Adjuvant chemotherapy, + (%) | 36 | (58) |

CEA, carcinoembryonic antigen; SUV, standardized uptake value; LN, lymph node; EGFR, epidermal growth factor receptor.

**Table S3.** Univariate and multivariate analyses for recurrence-free survival in pN2 patients.

|  | Univariate | | |
| --- | --- | --- | --- |
| Variable | HR | 95% CI | *P* |
| Age |  |  |  |
| +1 | 1.010 | 0.982 – 1.039 | 0.491 |
| Sex, male |  |  |  |
| female | ref. |  |  |
| male | 1.257 | 0.718 – 2.200 | 0.424 |
| Smoking history |  |  |  |
| never | ref. |  |  |
| ever | 1.244 | 0.711 – 2.176 | 0.445 |
| CEA |  |  |  |
| +1ng/ml | 1.002 | 0.997 – 1.007 | 0.468 |
| SUV max |  |  |  |
| primary, +1 | 1.062 | 0.994 – 1.136 | 0.076 |
| LN, +1 | 1.123 | 1.018 – 1.239 | 0.021 |
| Invasive size |  |  |  |
| +1cm | 1.084 | 0.925 – 1.271 | 0.319 |
| Lymphatic invasion |  |  |  |
| absent | ref. |  |  |
| present | 1.094 | 0.628 – 1.904 | 0.751 |
| Pleural invasion |  |  |  |
| absent | ref. |  |  |
| present | 1.520 | 0.867 – 2.664 | 0.143 |
| Tumor size in LN |  |  |  |
| +1mm | 1.049 | 0.994 – 1.106 | 0.080 |
| Number of N2 station |  |  |  |
| single | ref. |  |  |
| multiple | 1.603 | 0.918 – 2.799 | 0.097 |
| EGFR mutation |  |  |  |
| absent | ref. |  |  |
| present | 1.341 | 0.764 – 2.353 | 0.307 |
| Adjuvant chemotherapy |  |  |  |
| absent | ref. |  |  |
| present | 0.590 | 0.339 – 1.029 | 0.063 |

HR, hazard ratio; CI, confidence interval; ref., reference; CEA, carcinoembryonic antigen; SUV, standardized uptake value; LN, lymph node; EGFR, epidermal growth factor receptor.

**Table S4.** Clinicopathological differences in primary tumor between LN SUV max high and low.

| Characteristic | LN SUVmax low (<2.46)  N=31 | | | LN SUVmax high (≥2.46)  N=31 | | *P* |
| --- | --- | --- | --- | --- | --- | --- |
| SUV max, median [range] | 6.69 | [1.69 – 18.11] | 9.88 | | [2.66 – 17.48] | 0.050 |
| Invasive size, cm, median [range] | 2.3 | [0.9 – 7.8] | 3.0 | | [1.0 – 8.0] | 0.023 |
| Lymphatic invasion (%) |  |  |  | |  |  |
| absent | 16 | (52) | 18 | | (58) | 0.799 |
| present | 15 | (48) | 13 | | (42) |  |
| Vascular invasion (%) |  |  |  | |  |  |
| absent | 0 | (0) | 0 | | (0) | N/A |
| present | 31 | (100) | 31 | | (100) |  |
| Pleural invasion (%) |  |  |  | |  |  |
| absent | 18 | (58) | 9 | | (29) | 0.040 |
| present | 13 | (42) | 22 | | (71) |  |
| EGFR mutation (%), N=61 |  |  |  | |  |  |
| absent | 11 | (36) | 17 | | (57) | 0.195 |
| present | 19 | (63) | 13 | | (43) |  |
| Adjuvant chemotherapy (%) |  |  |  | |  |  |
| absent | 11 | (36) | 15 | | (48) | 0.440 |
| present | 20 | (65) | 16 | | (52) |  |

LN, lymph node; SUV, standardized uptake value; N/A, not applicable; EGFR, epidermal growth factor receptor.
